# Correction: High voltage DC power supply with power factor correction based on LLC resonant converter

**DOI:** 10.1371/journal.pone.0244595

**Published:** 2020-12-21

**Authors:** Muhammad Abid, Fiaz Ahmad, Farman Ullah, Usman Habib, Saeed Nawaz, Mohsin Iqbal, Ajmal Farooq

There are errors in the author affiliations. The correct affiliations are as follows:

Muhammad Abid^1^, Fiaz Ahmad^2^, Farman Ullah^1^, Usman Habib^3^, Saeed Nawaz^1^, Mohsin Iqbal^4*^, Ajmal Farooq^5^

**1** Department of Electrical Engineering, COMSATS Institute of Information Technology, Attock, Pakistan, **2** Department of Electrical & Computer Engineering, Air University, Islamabad, Pakistan, **3** Department of Computer Science, National University of Computer & Emerging Sciences, Islamabad, Pakistan, **4** Department of Electrical Engineering, Sarhad University of Science & IT, Peshawar, Pakistan **5** Department of Electrical Engineering, National University of Computer & Emerging Sciences, Islamabad, Pakistan,
