# Supplementary material for: High voltage DC power supply with power factor correction based on LLC resonant converter
Source: PLoS One. 2020 Sep 21;15(9):e0239008. doi: 10.1371/journal.pone.0239008 (PMC7505463; doi:10.1371/journal.pone.0239008)
Supplement: S1 File — (DOCX) [file pone.0239008.s001.docx]

**Meta data for reproduction of Simulation**

Various values used for generating the simulation results as well as the experimental results are given in Table 1.

Table 1: Circuit component values used in Simulation and experimental setup.

| S.No | Component | Value |
| --- | --- | --- |
| 1 | Vin | 220 Volts |
| 2 | Lf | 5mH |
| 3 | Lb | 200uH |
| 4 | C1&C2 | 330nF |
| 5 | Cbus | 10nF |
| 6 | Lm | 100uH |
| 7 | Cr | 30nF |
| 8 | Lr | 20uH |
| 9 | Co | 10nF |
| 10 | Switching frequency | 10 Hz |

**Software used for simulation:**

ORCAD Pspice was used as a simulation tool and all the simulation results were generated using this tool and the values given in Table 1.
