# Supplementary material for: High voltage DC power supply with power factor correction based on LLC resonant converter
Source: PLoS One. 2020 Sep 21;15(9):e0239008. doi: 10.1371/journal.pone.0239008 (PMC7505463; doi:10.1371/journal.pone.0239008)
Supplement: Attachment — Submitted filename: Response to Reviewer Comments.docx [file pone.0239008.s003.docx]

**Manuscript PONE-D-20-11491**

**Response to Reviewer(S)**

Dear Dr. Wang,

Thanks for giving us an opportunity to submit a revised version of manuscript entitled “High voltage DC power supply with power factor correction based on LLC Resonant Converter” for possible publication in your esteemed journal. We appreciate the time and effort put by you and reviewer(s). We have incorporated the changes required and these are highlighted in red color within the manuscript. Please see below in blue, point by point response to the reviewer(s) comments.

**Reviewer(s) Comments to Authors:**

**Reviewer 1:**

1. The Figure 1 is fuzzy.

Author(s) Response: Thank you for pointing out this, Fig. 1 has been redrawn and its quality is improved in revised manuscript

2. Authors should include references from recent years.

Author(s) Response: Most of the references have been updated with recent ones in the revised manuscript

Author(s) responses to other comments in the letter:

1. In your Data Availability statement, you have not specified where the minimal data set underlying the results described in your manuscript can be found. PLOS defines a study's minimal data set as the underlying data used to reach the conclusions drawn in the manuscript and any additional data required to replicate the reported study findings in their entirety. All PLOS journals require that the minimal data set be made fully available. Upon re-submitting your revised manuscript, please upload your study’s minimal underlying data set as either Supporting Information files or to a stable, public repository and include the relevant URLs, DOIs, or accession numbers within your revised cover letter.

Author(s) Response: A minimal set of data has now been added which can be used to regenerate the results depicted in the manuscript.

2. Please complete your Competing Interests on the online submission form to state any Competing Interests. If you have no competing interests, please state "The authors have declared that no competing interests exist."

Author(s) Response: Conflict of interest statement among authors added in the cover letter.

3. Please ensure that you refer to Figure 5 in your text as, if accepted, production will need this reference to link the reader to the figure.

   Author(s) Response: Figure 5 has now properly been referenced in the manuscript
4. Please upload a copy of Figure 12, to which you refer in your text on page 20. If the figure is no longer to be included as part of the submission please remove all reference to it within the text.

Author(s) Response: Figure 12 has now properly been referenced in the manuscript.
